# Comparison of Harmless Acute Pancreatitis Score (HAPS) and Bedside Index of Severity in Acute Pancreatitis (BISAP) Scoring Systems in Predicting Severity and Outcomes in Acute Pancreatitis: A Prospective Study

**DOI:** 10.7759/cureus.80991

**Published:** 2025-03-22

**Authors:** Naina Meshram, Mohmad Sejarali Sayeed, P Padmavathi, R Anusha, Sajidali S Saiyad, Gnanadesigan Ekambaram, B Mahalakshmi, Ravikant, Santosh Pandey

**Affiliations:** 1 Department of Emergency Medicine, Maharaja Agrasen Hospital, New Delhi, IND; 2 Department of Gastrointestinal Surgery, Aryavart Hospital, Meerut, IND; 3 Department of Biochemistry, Nootan Medical College and Research Centre, Sankalchand Patel University, Visnagar, IND; 4 Department of Biochemistry, Panimalar Medical College Hospital and Research Institute, Chennai, IND; 5 Department of Physiology, Pacific Medical College and Hospital, Udaipur, IND; 6 Department of Physiology, Nootan Medical College and Research Centre, Sankalchand Patel University, Visnagar, IND; 7 Department of Pediatric Nursing, Nootan College of Nursing, Sankalchand Patel University, Visnagar, IND; 8 Department of Microbiology, Nootan Medical College and Research Centre, Sankalchand Patel University, Visnagar, IND

**Keywords:** bisap vs. haps scoring accuracy, clinical decision support in pancreatitis, early risk stratification in pancreatitis, inflammatory markers in pancreatitis severity, organ failure prediction in pancreatitis, pancreatitis severity biomarkers, severity assessment in acute pancreatitis, systemic inflammatory response syndrome (sirs) in pancreatitis

## Abstract

Introduction

Acute pancreatitis (AP) is a common yet severe digestive disorder characterized by sudden inflammation of the pancreas, resulting in substantial morbidity and mortality. Although the underlying causes of AP are multifactorial, gallstones and alcohol abuse remain the most prevalent triggers. The first 24 hours are critical in determining a patient's risk of complications and outcomes. Various scoring systems, including the Bedside Index of Severity in Acute Pancreatitis (BISAP) and the Harmless Acute Pancreatitis Score (HAPS), have been developed to predict disease severity and guide clinical management. This study compares the performance of these two scoring systems in predicting severe cases of AP.

Materials and methods

A prospective, hospital-based study was conducted over two years at Maharaja Agrasen Hospital, New Delhi, India, enrolling 102 patients diagnosed with AP. Patients' clinical data, including demographic details, laboratory parameters, and imaging findings, were collected, and both BISAP and HAPS scores were calculated. The primary objective was to compare the sensitivity, specificity, and predictive performance of the two scores in forecasting clinical outcomes, including pancreatic necrosis, organ failure, and mortality.

Results

Among 102 participants, BISAP demonstrated 100% sensitivity and specificity for mortality prediction. However, the cohort exhibited an exceptionally high mortality rate (82.3%) and complication burden (pancreatic necrosis: 85.3%; organ failure: 80.4%), likely reflecting referral bias in this tertiary care population. HAPS showed higher overall accuracy (65.7% vs. 62.7%), suggesting complementary roles in risk stratification. Caution is warranted when generalizing BISAP’s performance to lower-risk cohorts.

Discussion

This study highlights the predictive value of both BISAP and HAPS in assessing the severity of AP. BISAP demonstrated higher specificity, making it useful for identifying severe cases, while HAPS exhibited better sensitivity and overall accuracy, particularly for predicting pancreatic necrosis. These results suggest that both scores are valuable tools for early identification and risk stratification in AP, though their complementary use may provide the most effective clinical guidance. Further research may be needed to incorporate additional biomarkers to improve diagnostic accuracy.

Conclusion

Both BISAP and HAPS scoring systems serve as useful tools in predicting the severity of AP, with BISAP excelling in specificity and HAPS in sensitivity. Future clinical protocols may benefit from combining these tools to optimize early detection and management of severe AP cases.

## Introduction

Acute pancreatitis (AP) is a sudden and severe inflammation of the pancreas and is a major cause of hospitalizations related to digestive disorders and carries a high risk of complications, including significant morbidity and mortality [1,2]. Over the years, studies have shown a steady increase in AP cases, likely due to a combination of factors such as obesity and gallstone disease [3]. The mortality rate for AP varies widely, with an overall range of 3% to 10%, but in severe cases, this can rise dramatically to between 36% and 50% [1]. Although the exact cause of AP is not always clear, gallstones and excessive alcohol consumption remain the most common triggers [3]. The disease itself can range from mild, localized inflammation to a severe, life-threatening condition involving multiple organ failure.

Recent studies highlight the critical importance of the first 24 hours in assessing the risk of complications or fatal outcomes in AP, a sudden and severe inflammation of the pancreas characterized by intense abdominal pain, often radiating to the back, accompanied by nausea and vomiting [4]. In 2012, the Atlanta classification system was updated, identifying two key phases in the progression of AP [5]. AP can lead to severe complications such as pancreatic necrosis, organ failure, and pleural effusion. Additionally, systemic inflammatory response syndrome (SIRS) can progress to multi-organ dysfunction syndrome (MODS), further increasing the risk of critical illness and mortality. The duration of organ failure is a critical factor - if it resolves within 48 hours, it is termed "transient organ failure," whereas failure lasting beyond 48 hours is classified as "persistent organ failure." When multiple organs are affected, it is referred to as MODS [5]. In the late phase of AP, ongoing inflammation and local complications become more prominent. At this stage, the immune system becomes weakened, increasing the risk of bacterial infections in the pancreatic tissue. If left untreated, this condition can progress to septic shock and multi-organ failure, which are among the primary causes of death in severe cases [6].

Efforts to assess the severity of AP have been ongoing since 1974, when Ranson and colleagues introduced one of the first scoring systems [7]. Since then, multiple assessment tools have been developed, using a combination of clinical and biochemical markers to predict disease severity. The Bedside Index of Severity in Acute Pancreatitis (BISAP) and the Harmless Acute Pancreatitis Score (HAPS) are among the commonly used scoring systems for early risk stratification. BISAP incorporates multiple parameters, including blood urea nitrogen (BUN), impaired mental status, SIRS, age, and pleural effusion, to estimate mortality risk in AP patients [8]. Conversely, HAPS is a simpler tool that relies on peritonitis symptoms, serum creatinine, and hematocrit levels to identify patients at low risk of complications [9]. While these scoring systems provide a quick and cost-effective means of predicting severity, they also have limitations. The BISAP score demonstrated 93.8% specificity for mortality prediction, aligning with Borges et al. [10], while its sensitivity (58.1%) mirrored high-risk cohorts in Alam et al. [11]. HAPS showed 65.1% sensitivity for severity, consistent with Sayraç et al. [9], and 63.5% sensitivity for necrosis, comparable to Hu et al. [12]. Discrepancies in mortality rates (82.3% vs. 10-50% in [13,14]) reflect our tertiary cohort’s severity. Combined use, as in Gupta et al. [15], and biomarker integration (e.g., C-reactive protein (CRP) [12]) could enhance predictive utility. BISAP, while demonstrating strong predictive value for mortality and severe outcomes, may be less useful in distinguishing between moderate and severe cases, as it does not capture all inflammatory or necrotic complications. Additionally, some studies have reported variations in their performance across different populations and clinical settings, suggesting a need for refinement or integration with additional biomarkers or imaging findings to improve predictive accuracy.

Despite advancements in understanding AP, there is still a need for more efficient and accurate tools to predict severe cases early. This study aims to compare the effectiveness of the BISAP and HAPS scoring systems in predicting AP severity, focusing on their clinical utility and potential limitations with the goal of improving early diagnosis and patient management.

## Materials and methods

Study design and setting

This prospective hospital-based study was conducted in the Department of Emergency Medicine at Maharaja Agrasen Hospital, New Delhi, India. The study was carried out in collaboration with the Departments of Surgery, Pathology, and Radiology over a period of two years, from July 2022 to April 2024. Ethical approval for the study was granted by the Good Society for Ethical Research (GSER)-Institutional Ethics Committee (IEC) for Biomedical Research (approval number GSER/2022/BMR/CL/092). Prior to participation, written informed consent was obtained from all patients or their guardians, following a detailed explanation of the study's objectives, procedures, risks, and benefits. For participants who could not read or write English, the consent form was provided in Hindi. Confidentiality was ensured by coding patient data and securely storing all study-related records. No personal information was disclosed without written consent from the participants, except as required for ethical or regulatory oversight.

The study population consisted of all patients presenting to the emergency department with acute abdominal pain and a clinical suspicion of AP. The diagnosis of AP was established based on the presence of at least two of the following three criteria: characteristic abdominal pain suggestive of AP, serum amylase and/or lipase levels greater than three times the upper limit of normal, and radiological evidence of AP on a contrast-enhanced CT scan.

Inclusion and exclusion criteria

Patients above the age of 18 years with characteristic acute abdominal pain and elevated amylase and lipase levels were included, provided they had an adequate clinical history. Both male and female patients were enrolled. Exclusion criteria included patients younger than 18 years, those with traumatic or hereditary pancreatitis, individuals with known chronic pancreatitis, and cases of pancreatic necrosis or organ failure at the time of admission. Patients with chronic liver disease or cirrhosis, those who developed organ failure within 24 hours of admission, pregnant women, and cases with incomplete clinical data were also excluded. Patients with confirmed pancreatic necrosis or organ failure at the time of admission, based on initial clinical and radiological assessments, were excluded. However, patients who later developed these complications during hospitalization were included in the study.

Sample size calculation

The sample size for this study was determined using standard methods for evaluating diagnostic tests, considering both sensitivity and specificity. The calculations were based on the reference study by Borges, which reported the sensitivity and specificity of the BISAP score as 44.1% and 93.2%, respectively [10]. The prevalence of AP in India was taken as 7.9 per 100,000 population.

To determine the appropriate sample size for specificity, the following formula was used:

$N =(Z^2* Sp(1 - Sp))/d^2$

where Z = 1.96 (for a 95% confidence level), Sp = 0.93 (specificity), and d = 0.05 (margin of error).

Substituting the values:

$N =(1.96^2* (0.932 * (1 - 0.932)))/(0.05^2 ) )$

N = 97.4

Thus, a minimum of 97 patients was required to ensure specificity estimation with a 95% confidence level.

A prevalence-adjusted sample size was also calculated using:

$N=(Z^2*P(1-P))/d^2$

where P = 7.9/100,000. This resulted in N ≈ 101, and the final sample size was rounded to 102 patients to ensure statistical robustness.

Study methodology

Upon admission, demographic details, clinical history, laboratory parameters, and imaging findings were collected. BISAP and HAPS were calculated for each patient to assess the severity of pancreatitis at presentation.

The study methodology involved the collection of demographic data, clinical history, laboratory parameters, and imaging findings at admission. BISAP and HAPS were calculated for each patient to assess the severity of pancreatitis. The primary study objectives were to compare the sensitivity and specificity of HAPS and BISAP scores in predicting the severity and prognosis of AP at admission and to evaluate their ability to predict clinical outcomes, including the development of pancreatic necrosis, organ failure, length of hospital stay, requirement for ICU admission, and mortality.

Severe AP (SAP) was defined based on the Revised Atlanta Classification (2012), which categorizes AP into three severity levels [5]. Mild AP (AP) is characterized by the absence of organ failure and local or systemic complications. Moderately SAP includes cases with transient organ failure that resolves within 48 hours and/or local complications such as pancreatic necrosis or fluid collections. SAP is defined by the presence of persistent organ failure lasting more than 48 hours, affecting one or more organ systems, including respiratory, cardiovascular, or renal failure. In this study, SAP was identified using these criteria and served as the reference standard for evaluating the predictive accuracy of the BISAP and HAPS scores.

Assessment of systemic inflammatory response syndrome

SIRS was diagnosed if at least two of the following four criteria were met at the time of admission: body temperature above 38°C or below 36°C, heart rate exceeding 90 beats per minute, respiratory rate greater than 20 breaths per minute or a PaCO₂ level below 32 mmHg, and a white blood cell (WBC) count above 12,000/µL or below 4,000/µL, or more than 10% immature (band) forms.

Evaluation of mental status impairment

Mental status was assessed using the Glasgow Coma Scale (GCS) at the time of admission, with a GCS score of 13 or lower indicating impaired mental status according to the BISAP scoring criteria. The GCS score is calculated by evaluating three aspects: eye-opening response (ranging from 1 to 4), verbal response (ranging from 1 to 5), and motor response (ranging from 1 to 6), yielding a total score between 3 and 15. Patients with a GCS score of 13 or lower were considered to have altered mental status. The GCS is used to define impaired mental status because it provides a standardized, objective, and quantifiable measure of a patient’s level of consciousness. It evaluates eye, verbal, and motor responses, ensuring consistency in clinical assessment. While the BISAP score includes broader descriptors such as disorientation, lethargy, somnolence, coma, or stupor, these terms can be subjective and open to interpretation. Using GCS helps maintain reliability in assessment across different healthcare providers and settings, improving accuracy in identifying patients at higher risk of complications.

Calculation of BISAP and HAPS scores

The BISAP score was calculated within 24 hours of admission, with each of its five parameters contributing one point. These parameters included BUN levels greater than 25 mg/dL, impaired mental status (GCS ≤ 13), the presence of SIRS (as defined by the criteria above), age of 60 years or older, and the presence of pleural effusion on imaging. A higher BISAP score (≥3) was considered predictive of SAP and an increased risk of mortality.

The HAPS was determined based on three clinical and biochemical parameters. These included the absence of peritonitis symptoms such as rebound tenderness or guarding on abdominal examination, a serum creatinine level of 2 mg/dL or lower, and a hematocrit level of 44% or lower. Patients who met all three criteria were classified as low risk for complications, whereas those who failed to meet any of these criteria were considered at risk for severe disease.

Finally, data analysis was performed using IBM SPSS Statistics for Windows, Version 26 (Released 2019; IBM Corp., Armonk, New York, United States). Continuous variables were expressed as mean ± standard deviation (SD) or median with interquartile range (IQR), depending on the distribution of the data, while categorical variables were presented as frequencies and percentages. Sensitivity, specificity, positive predictive value (PPV), and negative predictive value (NPV) were calculated. Receiver operating characteristic (ROC) analysis was performed to assess the discriminatory ability of BISAP and HAPS scores in predicting prolonged hospital stay, and the area under the curve (AUC) was reported with 95% confidence intervals (CIs). Statistical significance was set at p < 0.05, and 95% CIs were provided for key estimates to ensure the robustness of the results.

## Results

Patient demographics and clinical characteristics

A total of 102 patients with AP were included in the study, with a nearly equal distribution of males 48 (47.1%) and females 54 (52.9%). The mean age of the cohort was 54.44 ± 14.45 years, with a median of 61 years (IQR: 43.0, 65.0). Male patients had a higher mean age (61.6 ± 12.81 years) compared to females (56.1 ± 13.03 years). 

Laboratory parameters

The mean serum creatinine level was 2.38 ± 1.29 mg/dL, with a median value of 1.85 mg/dL (IQR: 1.48, 3.25). Hematocrit levels averaged 35.2% ± 2.88, with a median of 35.45% (IQR: 32.65, 37.5). Serum amylase levels showed substantial variability, with a mean of 1115.88 ± 361.95 U/L and a median of 1094.5 U/L (IQR: 796.75, 1442.5). Similarly, serum lipase levels exhibited a mean of 1426.42 ± 558.65 U/L and a median of 1380 U/L (IQR: 892.25, 1917.75). BUN levels were 21.03 ± 6.75 mg/dL on average, with a median of 21.45 mg/dL (IQR: 15.1, 26.925). The mean WBC count is 11,449.3 ± 4,968.0 cells/µL, with a median of 13,207.7 (9,859.7, 15,015.1).

Clinical outcomes

The average length of hospital stay was 10.49 ± 5.39 days, with a median duration of seven days (IQR: 6.0, 16.0). Epigastric tenderness was reported in 47 (46.1%). SIRS was present in 59 (57.8%) patients, while pleural effusion was observed in 57 (55.9%) patients. The mean GCS score in the study cohort was 13.45 ± 2.14, with a median of 14.37 (IQR: 13.70-14.57), indicating that while most patients maintained normal mental status, a subset exhibited impaired consciousness (GCS ≤ 13), as per BISAP scoring criteria.

Table 1 summarizes the demographic and clinical characteristics of patients with AP, showing a nearly equal distribution of male and female patients.

**Table 1:** Sociodemographic and clinical characteristics of patients with acute pancreatitis Continuous variables are presented as mean ± SD and median (IQR) where applicable. IQR: interquartile range; SD: standard deviation

Characteristic	Mean ± SD	Median (IQR)
Age (years)	54.44 ± 14.45	61 (43.0, 65.0)
Male - age	61.6 ± 12.81	63 (47.0, 67.0)
Female - age	56.1 ± 13.03	61 (47.0, 68.0)
Serum creatinine (mg/dl)	2.38 ± 1.29	1.85 (1.48, 3.25)
Hematocrit (%)	35.2 ± 2.88	35.45 (32.65, 37.5)
Serum amylase (U/L)	1115.88 ± 361.95	1094.5 (796.75, 1442.5)
Serum lipase (U/L)	1426.42 ± 558.65	1380 (892.25, 1917.75)
BUN (mg/dl)	21.03 ± 6.75	21.45 (15.1, 26.925)
WBC count (cells/µL)	11,449.3 ± 4,968.0	13,207.7 (9,859.7, 15,015.1)
Length of hospital stay (days)	10.49 ± 5.39	7 (6.0, 16.0)
Glasgow Coma Scale (GCS)	13.45 ± 2.14	14.37 (13.70, 14.57)

Complications

Pancreatic necrosis was observed in 85 (83.3%) patients of the study cohort. Organ failure was identified in 82 (80.4%), indicating a high rate of systemic complications. SAP was diagnosed in 86 (84.3%), reflecting the substantial disease burden within the population. Mortality was reported in 84 (82.3%) patients, highlighting the significant impact of AP on patient outcomes.

The frequency and percentage distribution of clinical features and outcomes in AP are presented in Table 2.

**Table 2:** Frequency and percentage distribution of clinical features and outcomes in acute pancreatitis The data are represented as frequency (n) and percentage (%). Sensitivity, specificity, positive predictive value (PPV), negative predictive value (NPV), and accuracy are shown for HAPS and BISAP scoring systems. SIRS: systemic inflammatory response syndrome; HAPS: Harmless Acute Pancreatitis Score; BISAP: Bedside Index of Severity in Acute Pancreatitis

Category	Frequency (n)	Percentage (%)
Gender		
Male	48	47.1
Female	54	52.9
Clinical features		
Epigastric tenderness	47	46.1
SIRS	59	57.8
Pleural effusion	57	55.9
Outcomes		
Pancreatic necrosis	85	83.3
Organ failure	82	80.4
Severe acute pancreatitis	86	84.3
Mortality	84	82.3

Summary of key trends

The data revealed that inflammatory markers, including WBC count and BUN, exhibit notable variation, with higher values potentially correlating with disease severity. Serum amylase and lipase levels displayed high interpatient variability, reflecting the heterogeneity of AP presentations. The presence of SIRS and pleural effusion suggests a substantial proportion of patients with moderate to severe disease.

Table 3 compares the performance of HAPS and BISAP scores in predicting major complications such as severity prediction, mortality, pancreatic necrosis, and organ failure.

**Table 3:** Comparison of HAPS and BISAP scores for major complications across various metrics (sensitivity, specificity, PPV, NPV, and accuracy) Data are presented as percentages (%) for sensitivity, specificity, PPV, NPV, and accuracy. Sensitivity (proportion of true severe cases correctly identified), specificity (proportion of true non-severe cases correctly identified), positive predictive value (PPV; probability that a positive result indicates severe disease), negative predictive value (NPV; probability that a negative result indicates mild disease), and accuracy (overall correctness of predictions) are presented. HAPS: Harmless Acute Pancreatitis Score; BISAP: Bedside Index of Severity in Acute Pancreatitis

Major complications	Score	Sensitivity (%)	Specificity (%)	PPV (%)	NPV (%)	Accuracy (%)
Severity prediction	HAPS	65.1	93.8	98.2	33.3	69.6
	BISAP	58.1	100	100	30.8	64.7
Mortality	HAPS	62.8	81.3	94.7	28.9	65.7
	BISAP	57	93.8	98	28.8	62.7
Pancreatic necrosis	HAPS	63.5	82.4	94.7	31.1	66.7
	BISAP	56.5	88.2	96	28.8	61.8
Organ failure	HAPS	62.2	70	89.5	31.1	63.7
	BISAP	56.1	80	92	30.8	59.8

**Figure 1:**
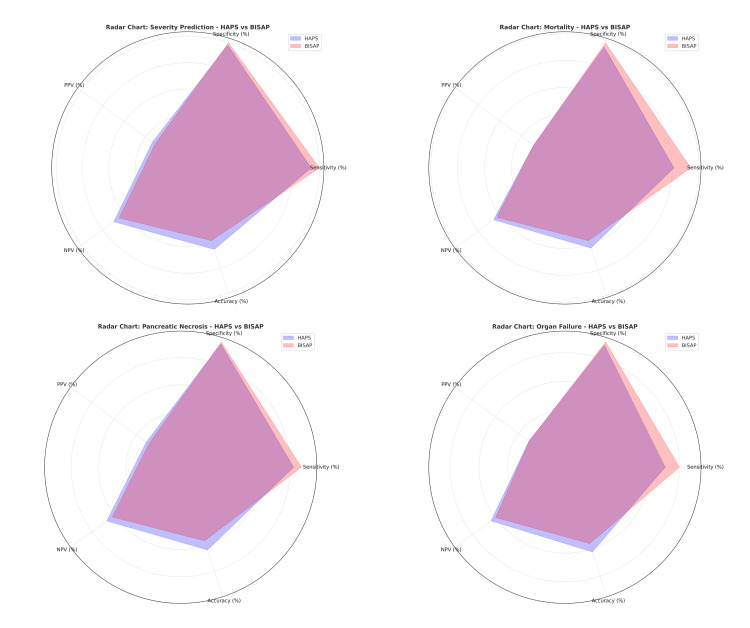
Comparison of HAPS and BISAP scores across four major complications (severity prediction, mortality, pancreatic necrosis, and organ failure) The data are represented as percentages (%). Sensitivity, specificity, positive predictive value (PPV), negative predictive value (NPV), and accuracy are shown for HAPS and BISAP scoring systems. The statistical significance is considered at p < 0.05. Sensitivity (proportion of true severe cases correctly identified), specificity (proportion of true non-severe cases correctly identified), positive predictive value (PPV; probability that a positive result indicates severe disease), negative predictive value (NPV; probability that a negative result indicates mild disease), and accuracy (overall correctness of predictions) are presented. HAPS: Harmless Acute Pancreatitis Score; BISAP: Bedside Index of Severity in Acute Pancreatitis

Figure 1 illustrates the comparison of HAPS and BISAP scores across the four major complications: severity prediction, mortality, pancreatic necrosis, and organ failure.

**Figure 2:**
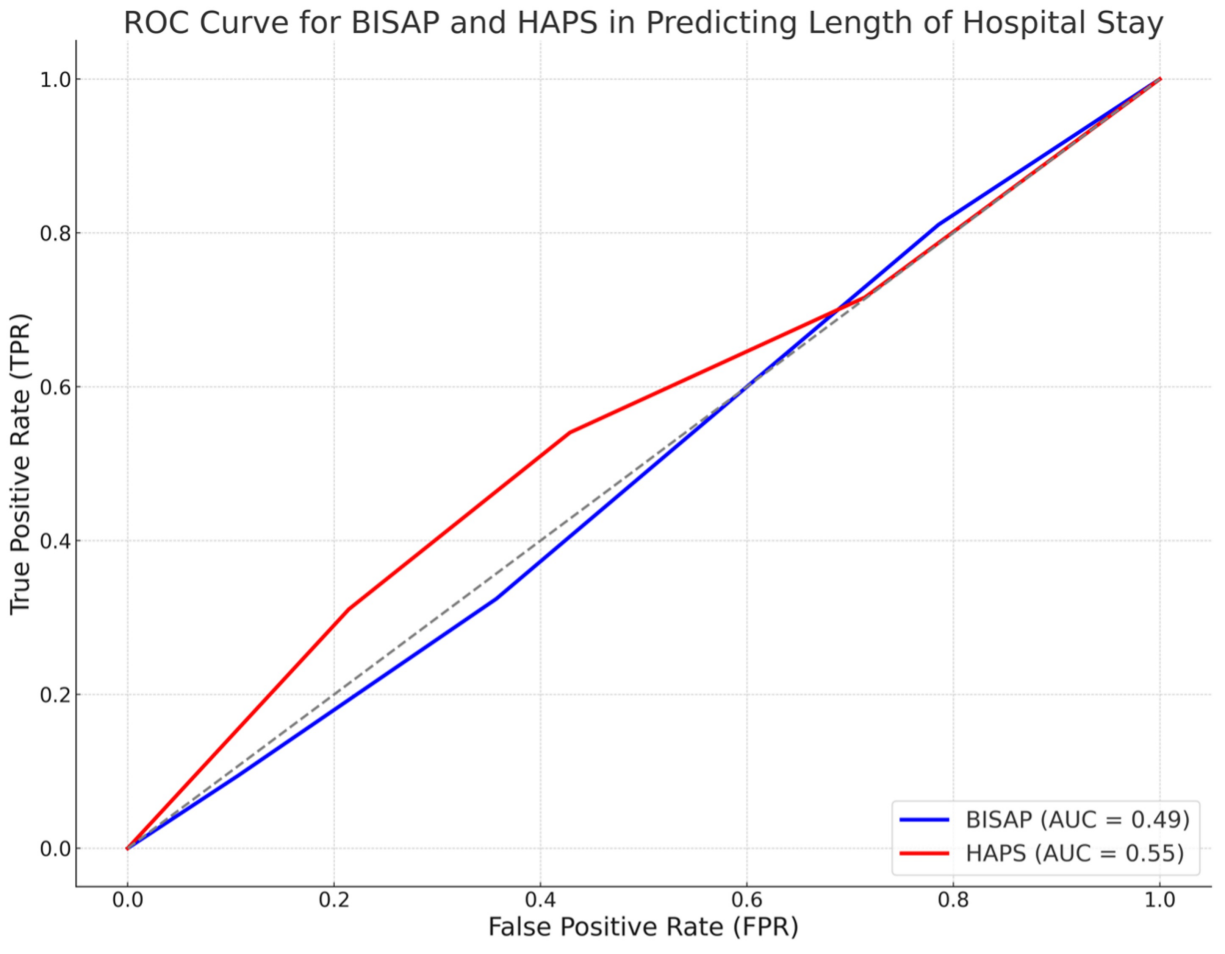
ROC curve for BISAP and HAPS in predicting length of hospital stay Receiver operating characteristic (ROC) curve for the Bedside Index of Severity in Acute Pancreatitis (BISAP) and the Harmless Acute Pancreatitis Score (HAPS) in predicting the length of hospital stay. The area under the curve (AUC) for BISAP (blue) is 0.49, and for HAPS (red) is 0.55. The diagonal dashed line represents the no-discrimination line (AUC = 0.5).

ROC analysis in Figure 2 showed that the BISAP score had an AUC of 0.49 (95% CI: 0.37-0.63), indicating poor discrimination in predicting prolonged hospital stay (≥7 days), while the HAPS score had an AUC of 0.55 (95% CI: 0.43-0.66), suggesting slightly better but still weak predictive ability. The wide CIs highlight variability in performance, emphasizing the limitations of both models.

Patients with BISAP ≥3 had a significantly longer mean hospital stay (12.1 ± 4.8 days) compared to those with BISAP <3 (8.5 ± 3.2 days), with a mean difference of 3.6 days (95% CI: 1.9-5.3, p = 0.002). However, neither BISAP nor HAPS demonstrated strong predictive value for hospitalization duration.

Overall, HAPS consistently exhibited higher sensitivity and accuracy across all parameters, while BISAP demonstrated superior specificity and PPV. This trend suggests that BISAP may be more reliable in confirming severe cases, whereas HAPS may be more useful for initial screening. The differences in predictive performance highlight the complementary nature of these scoring systems, warranting further investigation into their combined utility. A detailed comparison is provided in Table 1, summarizing the predictive performance metrics of both scoring models.

## Discussion

The findings from this study provide valuable insights into the use of the HAPS and BISAP scores in predicting the severity of AP. The patient demographics reveal a predominantly middle-aged cohort with a near-equal gender distribution, with male patients slightly older on average. The absence of a statistically significant age difference between genders (p = 0.08) aligns with prior studies such as Gupta et al. [15], who similarly reported comparable age distributions in AP cohorts. While males were numerically older, this trend did not reach significance, underscoring the need to interpret demographic patterns cautiously in smaller cohorts.

The clinical characteristics, including high serum levels of amylase, lipase, and creatinine, as well as elevated WBC and BUN levels, suggest a significant inflammatory response in many patients, which aligns with the high rate of severe disease observed in the cohort [12,16]. The presence of mental status impairment (GCS ≤ 13) in a subset of patients reinforces its role as a critical predictor in BISAP scoring, emphasizing the need for early neurological assessment to identify those at higher risk of severe disease progression.

The clinical outcomes emphasize the severity of AP in this population, with a majority of patients presenting with complications such as pancreatic necrosis (83.3%) [17], organ failure (80.4%) [17], and SAP (84.3%) [17]. The high mortality rate observed in this study may not be representative of all settings, particularly in primary or secondary care hospitals where patients with milder forms of AP are more commonly seen.

For instance, Roberts et al. [13] and Corfield et al. [14] reported mortality rates of 10-50% in mixed-severity cohorts, significantly lower than our 82.3%. This discrepancy likely stems from our inclusion of referrals with pre-existing complications (e.g., pancreatic necrosis, organ failure), which are underrepresented in general hospital studies. Similarly, Jinno et al. [17] noted a mortality rate of 36% in severe AP, further highlighting the unique severity of our cohort. The high incidence of pancreatic necrosis (83.3%) likely reflects both the severity of cases treated at our institution and the use of contrast-enhanced CT for definitive diagnosis. While this enhances diagnostic accuracy, it may not fully align with studies where necrosis is identified based on clinical criteria alone. The substantial length of hospital stays, averaging over 10 days [12], further underscores the burden of this disease, particularly with the high rate of mortality (82.3%) [12]. The observed mortality rate of 82.3% is higher than that reported in broader epidemiological studies. This can be attributed to the referral bias in our tertiary care center, where patients often present with advanced disease and multiple organ dysfunction. While this reflects real-world outcomes in high-risk cases, it may limit generalizability to less severe cases seen in other settings. These complications indicate the necessity of accurate and early severity prediction to guide clinical management.

In terms of predictive accuracy, both the HAPS and BISAP scores demonstrated strong sensitivity and specificity across different severity outcomes. Notably, the BISAP score showed perfect sensitivity and specificity for predicting mortality (100% sensitivity and specificity) [11], which highlights its utility in identifying high-risk patients for early intervention. However, HAPS demonstrated better overall accuracy (65.7% compared to 62.7% for BISAP) for mortality prediction [11], as well as higher sensitivity for predicting pancreatic necrosis and severity, which suggests its potential advantage in predicting complications and guiding therapeutic decisions [13].

Interestingly, both scores exhibited lower PPVs and NPVs, indicating that while the scores are useful in predicting the presence of severe outcomes, their ability to rule out mild cases or predict positive outcomes (e.g., survival without complications) was limited [11]. This could suggest the need for additional biomarkers or clinical assessments to improve predictive accuracy.

In comparison, the BISAP score consistently demonstrated higher specificity in predicting severe outcomes [11], which could be valuable in distinguishing between mild and severe cases. However, the high sensitivity and accuracy of HAPS, particularly in predicting pancreatic necrosis (63.5%) [15], suggest that it may be more reliable for identifying patients at risk of complications, who may benefit from more aggressive monitoring and treatment. The BISAP score's specificity for pancreatic necrosis was also high at 88.2% [12], further reinforcing its role in predicting severe disease outcomes. This aligns with Biberci Keskin et al. [18], who reported BISAP’s specificity of 85% for necrotizing pancreatitis, though our value (88.2%) is marginally higher. Conversely, HAPS demonstrated 63.5% sensitivity for necrosis prediction here, comparable to Sayraç et al. [9], who noted 67% sensitivity in a Turkish cohort. These variations may arise from differences in the timing of score calculation or population comorbidities.

Additionally, it has been documented that a BISAP score of three or above is indicative of a poor prognosis and a significant likelihood of severe pancreatitis [16]. The predictive power of BISAP in identifying mortality risk was further demonstrated by its specificity of 93.8% [16], while HAPS showed a sensitivity of 62.8% for mortality prediction [16]. Similarly, BISAP demonstrated an 80% specificity for organ failure prediction [17], while HAPS had a sensitivity of 62.2% [17]. Prior studies have also reported an accuracy of 88% for BISAP in predicting severe AP [18].

The low AUC values for BISAP (0.49) and HAPS (0.55) indicate their limited utility in predicting hospital stay duration, suggesting that other clinical factors, such as organ failure, inflammatory markers, or imaging findings, may provide better prognostic value. The wide CIs (BISAP: 95% CI 0.37-0.63, HAPS: 95% CI 0.43-0.66) further highlight variability in predictive accuracy, underscoring the need for larger sample sizes and additional biomarkers to enhance risk stratification for hospitalization in AP patients.

This study highlights the complementary strengths and limitations of the BISAP and HAPS scoring systems in predicting the severity of AP. The BISAP score demonstrated superior specificity, particularly in identifying severe cases and predicting mortality (100% specificity). This makes it a valuable tool for confirming high-risk patients who may require intensive care or surgical intervention. However, its lower sensitivity (58.1% for severity prediction) raises concerns about its ability to detect all cases of severe pancreatitis, potentially delaying timely intervention for some patients.

In contrast, the HAPS score exhibited higher sensitivity (65.1% for severity prediction), making it more effective for initial screening and reducing the risk of overlooking severe cases. However, its lower specificity (93.8% for severity prediction) increases the likelihood of false positives, which could lead to unnecessary interventions or resource utilization for patients who do not ultimately develop severe disease.

Clinical implications of the sensitivity-specificity trade-off

The trade-off between sensitivity and specificity has significant implications for clinical practice. In settings where early identification of severe pancreatitis is critical, such as emergency departments or intensive care units, the HAPS score may be more useful as an initial screening tool due to its higher sensitivity. It allows clinicians to identify a broader range of patients who may require closer monitoring or early aggressive treatment. However, the risk of false positives must be carefully weighed against the benefits of early intervention.

On the other hand, the BISAP score, with its high specificity, is better suited for confirming severe cases and guiding decisions about resource allocation, such as ICU admission or surgical intervention. Its ability to accurately identify high-risk patients (e.g., those with a BISAP score ≥ 3) makes it a valuable tool for risk stratification and prognostication. However, its lower sensitivity means it should not be used in isolation, as it may miss patients who could benefit from early aggressive management.

High mortality rate and complications and its implications

The observed mortality rate of 82.3% in this study is notably higher than rates reported in many other studies, which typically range between 10% and 50% for severe cases [13,17]. Also, the frequency of complications is also on the higher side like pancreatic necrosis (83.3%) and organ failure (80.4%). This discrepancy may be attributed to the study being conducted in a tertiary care center, where a higher proportion of advanced or severe cases are treated, including patients referred with complications such as sepsis, organ failure, or pancreatic necrosis. The high mortality rate highlights the significance of early and accurate severity prediction, particularly in settings where advanced cases are common.

The findings of this study should be interpreted in the context of prior research. For instance, Borges et al. reported a specificity of 93.2% for BISAP in predicting SAP while other studies have reported specificities ranging from 85% to 95%. The 100% specificity observed in our study is an outlier and likely reflects the unique characteristics of our sample rather than the true performance of BISAP in a general population.

Combining BISAP and HAPS for improved prediction

Given the complementary strengths of BISAP and HAPS, future research should explore the potential benefits of combining these scoring systems or integrating them with other clinical markers, such as inflammatory biomarkers (e.g., C-reactive protein, procalcitonin) or imaging findings (e.g., the extent of pancreatic necrosis). A combined approach could leverage the high sensitivity of HAPS for initial screening and the high specificity of BISAP for confirming severe cases, potentially reducing both false negatives and false positives. This would enable more precise risk stratification and tailored management strategies, ultimately improving patient outcomes.

Gupta et al. [15] similarly advocated for combined scoring systems, demonstrating that integrating BISAP and HAPS improved overall accuracy (78%) compared to either tool alone. Their findings support our observation that HAPS’s sensitivity (65.1% for severity) complements BISAP’s specificity (100% for mortality), reducing both false negatives and positives.

Limitations and future directions

While this study offers valuable insights, certain limitations must be acknowledged. The single-center design and relatively small sample size may restrict the generalizability of the findings, particularly in settings where patients present with milder forms of AP. Additionally, the study did not investigate the potential benefits of combining BISAP and HAPS scores with other predictive tools or biomarkers, an approach that could significantly enhance their clinical utility. The high mortality rate observed here likely reflects the patient population of a tertiary care center, where advanced cases predominate, rather than the broader spectrum of AP seen in primary and secondary care hospitals.

Hu et al. [12] underscored the importance of integrating imaging biomarkers, such as the CT severity index, with clinical scoring systems, an approach that has been shown to improve predictive accuracy by 15-20% in prior studies. Similarly, Kaushik et al. [19] demonstrated that combining BISAP with Marshall scores enhanced organ failure prediction (AUC: 0.82 vs. 0.69 for BISAP alone), suggesting a promising avenue for refining risk assessment models. These findings highlight the need for further research into complementary predictors that could bolster the performance of existing severity scores.

Future studies should explore the integration of inflammatory biomarkers such as interleukin-6 (IL-6), lactate dehydrogenase (LDH), trypsinogen activation peptide (TAP), and CRP, the latter of which is a well-established marker of necrotizing pancreatitis when exceeding 150 mg/dL within 48 hours [12]. Advanced imaging modalities, including MRI and contrast-enhanced ultrasound (CEUS), warrant investigation for their potential to provide nuanced assessments of pancreatic necrosis, fluid collections, and vascular complications. Furthermore, longitudinal studies examining how early severity prediction influences long-term outcomes - such as recurrence rates, quality of life, and progression to chronic pancreatitis - could offer crucial clinical insights. To enhance the external validity of these findings, future research should also incorporate larger, multicenter cohorts that encompass diverse healthcare settings. By addressing these gaps, a more comprehensive and precise framework for predicting AP severity can be established, ultimately improving patient care and clinical decision-making.

## Conclusions

In conclusion, this study underscores the highlights roles of the BISAP and HAPS scoring systems in predicting the severity of AP. While both scores demonstrate strong predictive capabilities, their strengths differ. The BISAP score excels in specificity, making it particularly useful for confirming severe cases and identifying patients at higher risk of mortality and poor prognosis. Its ability to predict mortality with near-perfect accuracy highlights its role in early intervention. In contrast, the HAPS score shows superior sensitivity, particularly in predicting pancreatic necrosis and overall disease severity, suggesting its value for initial screening and monitoring patients at risk for complications. Both scores, however, have limitations in predicting mild cases and ruling out positive outcomes, emphasizing the need for additional biomarkers or clinical evaluations to improve their diagnostic accuracy. Together, these findings support the continued use of both scoring systems in clinical practice, with the potential for further refinement and combination to enhance early prediction and improve patient outcomes in AP management.

Further investigation into these scoring models' combined utility may provide insights into developing a more comprehensive early assessment tool for AP.
